# Prevalence and Correlates of Long COVID Symptoms Among US Adults

**DOI:** 10.1001/jamanetworkopen.2022.38804

**Published:** 2022-10-27

**Authors:** Roy H. Perlis, Mauricio Santillana, Katherine Ognyanova, Alauna Safarpour, Kristin Lunz Trujillo, Matthew D. Simonson, Jon Green, Alexi Quintana, James Druckman, Matthew A. Baum, David Lazer

**Affiliations:** 1 Department of Psychiatry, Massachusetts General Hospital, Boston; 2 Department of Psychiatry, Harvard Medical School, Boston, Massachusetts; 3 Department of Political Science, Northeastern University, Boston, Massachusetts; 4 Department of Communication, School of Communication and Information, Rutgers University, New Brunswick, New Jersey; 5 John F. Kennedy School of Government and Department of Government, Harvard University, Cambridge, Massachusetts; 6 Department of Political Science, University of Pennsylvania, Philadelphia; 7 Department of Political Science, Northwestern University, Evanston, Illinois

## Abstract

**Importance:**

Persistence of COVID-19 symptoms beyond 2 months, or long COVID, is increasingly recognized as a common sequela of acute infection.

**Objectives:**

To estimate the prevalence of and sociodemographic factors associated with long COVID and to identify whether the predominant variant at the time of infection and prior vaccination status are associated with differential risk.

**Design, Setting, and Participants:**

This cross-sectional study comprised 8 waves of a nonprobability internet survey conducted between February 5, 2021, and July 6, 2022, among individuals aged 18 years or older, inclusive of all 50 states and the District of Columbia.

**Main Outcomes and Measures:**

Long COVID, defined as reporting continued COVID-19 symptoms beyond 2 months after the initial month of symptoms, among individuals with self-reported positive results of a polymerase chain reaction test or antigen test.

**Results:**

The 16 091 survey respondents reporting test-confirmed COVID-19 illness at least 2 months prior had a mean age of 40.5 (15.2) years; 10 075 (62.6%) were women, and 6016 (37.4%) were men; 817 (5.1%) were Asian, 1826 (11.3%) were Black, 1546 (9.6%) were Hispanic, and 11 425 (71.0%) were White. From this cohort, 2359 individuals (14.7%) reported continued COVID-19 symptoms more than 2 months after acute illness. Reweighted to reflect national sociodemographic distributions, these individuals represented 13.9% of those who had tested positive for COVID-19, or 1.7% of US adults. In logistic regression models, older age per decade above 40 years (adjusted odds ratio [OR], 1.15; 95% CI, 1.12-1.19) and female gender (adjusted OR, 1.91; 95% CI, 1.73-2.13) were associated with greater risk of persistence of long COVID; individuals with a graduate education vs high school or less (adjusted OR, 0.67; 95% CI, 0.56-0.79) and urban vs rural residence (adjusted OR, 0.74; 95% CI, 0.64-0.86) were less likely to report persistence of long COVID. Compared with ancestral COVID-19, infection during periods when the Epsilon variant (OR, 0.81; 95% CI, 0.69-0.95) or the Omicron variant (OR, 0.77; 95% CI, 0.64-0.92) predominated in the US was associated with diminished likelihood of long COVID. Completion of the primary vaccine series prior to acute illness was associated with diminished risk for long COVID (OR, 0.72; 95% CI, 0.60-0.86).

**Conclusions and Relevance:**

This study suggests that long COVID is prevalent and associated with female gender and older age, while risk may be diminished by completion of primary vaccination series prior to infection.

## Key Points

**Question:**

How common are COVID-19 symptoms lasting longer than 2 months, also known as *long COVID*, among adults in the United States, and which adults are most likely to experience long COVID?

**Findings:**

In this cross-sectional study of more than 16 000 individuals, 15% of US adults with a prior positive COVID-19 test reported current symptoms of long COVID. Those who completed a primary vaccination series prior to infection were less likely to report long COVID symptoms.

**Meaning:**

This study suggests that long COVID is prevalent and that the risk varies among individual subgroups in the United States; vaccination may reduce this risk.

## Teaser

This cross-sectional study of US adults estimates the prevalence of and sociodemographic factors associated with COVID-19 symptoms lasting beyond 2 months, or long COVID, and identifies whether the predominant variant at the time of infection and prior vaccination status are associated with differential risk.

## Introduction

For a subset of individuals with acute COVID-19 disease, symptoms may persist beyond 1 month, with some patients reporting symptoms at least 6 months later.^1^ Initially referred to as *postacute sequelae of COVID-19* or *post–COVID-19 syndrome*,^2^ this phenomenon is now more commonly described as *long COVID*.^3^ The World Health Organization^4^ defined long COVID as generally occurring 3 months from the onset of COVID-19 with symptoms that last for at least 2 months.

Prevalence estimates for long COVID vary widely, in part because of variability in the definition and sampling frame. A self-report symptom tracking study among 4182 individuals found rates of symptomatic persistence of 13.3% at 1 month and 4.5% at 2 months.^5^ In a United Kingdom COVID-19–focused survey, among 20 000 individuals with a positive SARS-CoV-2 test result, 13.7% reported symptom persistence at 12 weeks based on a single survey question.^6^

Two studies have used administrative claims or electronic health records to examine long COVID symptoms among samples not limited to inpatients. One investigation using administrative data from the Veterans Affairs health system in the United States confirmed that a range of symptoms affecting multiple organ systems was common, among them respiratory, metabolic, cardiovascular, gastrointestinal, and neuropsychiatric diagnoses,^7^ but did not report prevalence of the syndromes per se. More recently, a Centers for Disease Control and Prevention investigation using commercial electronic health record data found that 1 in 5 individuals aged 18 to 64 years and up to 1 in 4 individuals aged 65 years or older experienced new onset of a disease identified using a diagnostic code that could be associated with COVID-19 at or beyond 30 days from onset^8^; however, that study did not otherwise account for age, gender, or a range of other confounding features. A key limitation in both of these studies is the reliance on coded diagnoses, which may miss individual symptoms that do not contribute to a medical encounter or are not coded as part of an encounter; a study using natural language processing to identify such symptoms of long COVID reported prevalences of 10% to 15%.^9^

Numerous aspects of long COVID remain poorly understood, with reviews suggesting that this phenomenon may actually reflect multiple different syndromes.^3,10^ In particular, it is not known which individuals will experience full recovery and which individuals will experience persistence of symptoms. One such concern is whether disadvantaged groups, such as individuals from racial and ethnic minority groups or socioeconomically disadvantaged groups, may have a disproportionately high prevalence of long COVID because they have experienced a disproportionately high burden of acute infection.^11^ If high-risk individuals could be identified, it might be possible to develop strategies to mitigate or prevent symptom persistence, prompting calls for increased emphasis on investigation of postacute sequelae of COVID-19.^2,12^ A prior self-report study identified older age and female gender as correlates of greater risk for persistent COVID-19 symptoms^13^; associations with gender were further supported in a UK survey.^6^

A particular correlate of interest has been the role of prior vaccination and long COVID risk (ie, the extent to which so-called breakthrough infections might be associated with differential risk). In a recent reanalysis of Veterans Health Administration data,^14^ including more than 30 000 previously vaccinated individuals who experienced breakthrough infection, the risk of long COVID was modestly but statistically significantly diminished. A corresponding secondary analysis of a complementary study drew on self-report from more than 1 million users of a UK COVID-19 symptom tracking application, including approximately 8400 individuals who experienced COVID-19 infection after at least 1 vaccine dose^15^; that study found marked protection after a second vaccine dose but no significant protection from an initial dose.

To better characterize symptom persistence with a broader and less select sampling frame, we used data from a multiwave US survey that included questions about COVID-19 encompassing 50 states and the District of Columbia. The survey did not focus only on COVID-19, yielding less likelihood of selection bias than more focused surveys of COVID-19 persistence in which participants opt in. We aimed to characterize the prevalence of long COVID in the general US population, to identify sociodemographic features associated with persistence of symptoms for at least 2 months after onset, and to estimate the protective association, if any, of vaccination.

## Methods

### Study Design

We included data collected from 8 waves of the COVID States Project, a large-scale internet survey conducted for an academic consortium approximately every 6 weeks between February 5, 2021, and July 6, 2022, inclusive of all 50 states and the District of Columbia. Survey participants were individuals aged 18 years or older who resided in the United States. The nonprobability^16^ sampling method has previously been validated in similar contexts as a substantially lower-cost alternative to traditional survey approaches.^17,18^ The survey applied representative quotas to balance age, gender, race and ethnicity, and geographic distribution. Survey participants provided signed informed consent online prior to survey access. Because data were deidentified, the study was determined to be exempt by the institutional review board of Harvard University. This study followed the American Association for Public Opinion Research (AAPOR) reporting guideline.^19^

### Measures

All respondents were asked if they had received a positive COVID-19 test result, which did not distinguish between polymerase chain reaction test or antigen test, and in which month they received this result. Those who reported any positive diagnosis were further asked whether their symptoms had resolved; for those who identified continued symptoms, they were asked to complete a checklist of commonly reported symptoms. Month of first and second vaccination, where applicable, was also identified via a checklist. All sociodemographic variables were collected by self-report. Data on race and ethnicity were obtained from 5 US Census categories to confirm representativeness of the US population, with categories analyzed and reported in accordance with a recent medical publication guidance statement.^20^

### Statistical Analysis

We adapted the World Health Organization^4^ definition of long COVID, including all individuals whose survey start date was more than 2 months after the month in which they initially identified a positive COVID-19 test result and defining casees as reporting continued symptoms at the time of the survey. (A planned sensitivity analysis applied a stricter definition, excluding individuals who said that ongoing symptoms did not affect their life [answering “not at all” regarding effect], consistent with the World Health Organization reference to symptoms that “generally have an impact on everyday functioning,”^4^ and those for whom loss of smell was the only reported symptom). Vaccination prior to illness was defined by comparing the month of vaccination with the first identified month of illness. For purposes of primary analysis, completion of the primary vaccination series was defined as 2 vaccinations occurring prior to the first month of illness, or a single vaccination when the Ad.26.COV2.S vaccine (Janssen) was identified in response to the question, “Which COVID-19 vaccine did you receive?” The predominant US viral variant at the time of infection was derived on the basis of CoVariants analysis of GISAID (Global Initiative on Sharing Avian Influenza Data) data^21^ indicating 50% or more typed variants reflecting a given variant. For participants who responded to more than 1 survey wave, the most recent survey was included.

We applied multiple logistic regression in R, version 4.0 (R Project for Statistical Computing)^22^ to examine the association of persistence of symptoms with sociodemographic features, and then we extended these models to include terms for vaccination status and predominant variant at month of infection. Post hoc analysis also examined the association of age by decade to detect possible nonlinear associations. To generate population-weighted estimates of prevalence either among those with a prior positive COVID-19 test result or the adult US population as a whole regardless of COVID-19 status, survey results from all survey respondents were reweighted with interlocking national weights for age, gender, race and ethnicity, educational level, urbanicity (urban, suburban, or rural), and region, based on the 2019 US Census American Community Survey.^23^ All *P* values were from 2-sided tests and results were deemed statistically significant at *P* < .05

## Results

Without reweighting the survey sample, the 16 091 survey respondents reporting test-confirmed COVID-19 illness at least 2 months prior had a mean age of 40.5 (15.2) years; 10 075 (62.6%) were women, and 6016 (37.4%) were men; 817 (5.1%) were Asian, 1826 (11.3%) were Black, 1546 (9.6%) were Hispanic, and 11 425 (71.0%) were White. From this cohort, 2359 individuals (14.7%) reported continued COVID-19 symptoms more than 2 months after acute illness.

Table 1 summarizes additional characteristics of the resulting cohort, by presence or absence of persistent symptoms. When the cohort was restricted to the 12 441 individuals who tested positive for COVID-19 at least 6 months previously, 1843 (14.8%) reported continued COVID-19 symptoms. Of the 7462 individuals who tested positive at least 12 months previously, 1135 (15.2%) reported continued symptoms.

**Table 1.** Characteristics of Individuals Who Tested Positive for COVID-19 by Antigen Test or Polymerase Chain Reaction Test at Least 2 Months Prior to Survey Date

Characteristic	Individuals, No. (%)			*P* value
	Recovered (n = 13 732)	Long COVID (n = 2359)	Total (N = 16 091)	
Gender				
Male	5452 (39.7)	564 (23.9)	6016 (37.4)	<.001
Female	8280 (60.3)	1795 (76.1)	10 075 (62.6)	
Age, mean (SD), y	40.0 (15.2)	43.6 (15.2)	40.5 (15.2)	<.001
Educational level				
High school or less	3340 (24.3)	629 (26.7)	3969 (24.7)	<.001
Some college	4337 (31.6)	972 (41.2)	5309 (33.0)	
Bachelor’s degree	3580 (26.1)	520 (22.0)	4100 (25.5)	
Graduate degree	2475 (18.0)	238 (10.1)	2713 (16.9)	
Household income, $				
<25 000	3092 (22.5)	643 (27.3)	3735 (23.2)	<.001
25 000-74 999	5449 (39.7)	1058 (44.8)	6507 (40.4)	
75 000-149 999	3892 (28.3)	512 (21.7)	4404 (27.4)	
≥150 000	1299 (9.5)	146 (6.2)	1445 (9.0)	
Race and ethnicity				
Asian	751 (5.5)	66 (2.8)	817 (5.1)	<.001
Black	1595 (11.6)	231 (9.8)	1826 (11.3)	
Hispanic	1377 (10.0)	169 (7.2)	1546 (9.6)	
White	9615 (70.0)	1810 (76.7)	11 425 (71.0)	
Other race and ethnicity^a^	394 (2.9)	83 (3.5)	477 (3.0)	
Urbanicity				
Rural	2156 (15.7)	469 (19.9)	2625 (16.3)	<.001
Suburban	7615 (55.5)	1429 (60.6)	9044 (56.2)	
Urban	3961 (28.8)	461 (19.5)	4422 (27.5)	
Region				
Northeast	2171 (15.8)	315 (13.4)	2486 (15.4)	<.001
Midwest	3435 (25.0)	656 (27.8)	4091 (25.4)	
South	5329 (38.8)	958 (40.6)	6287 (39.1)	
West	2797 (20.4)	430 (18.2)	3227 (20.1)	
Vaccination status				
Unvaccinated	11 382 (82.9)	2052 (87.0)	13 434 (83.5)	<.001
Partial	356 (2.6)	58 (2.5)	414 (2.6)	
Complete	1994 (14.5)	249 (10.6)	2243 (13.9)	
Impact of symptoms				
Not at all	0	152 (6.4)	152 (6.4)	NA
A little	0	889 (37.7)	889 (37.7)	
Moderately	0	703 (29.8)	703 (29.8)	
Quite a bit	0	456 (19.3)	456 (19.3)	
Extremely	0	158 (6.7)	158 (6.7)	
Predominant variant				
Alpha	971 (7.1)	147 (6.2)	1118 (6.9)	<.001
Ancestral	7385 (53.8)	1344 (57.0)	8729 (54.2)	
Delta	2074 (15.1)	416 (17.6)	2490 (15.5)	
Epsilon	1351 (9.8)	206 (8.7)	1557 (9.7)	
Omicron	1951 (14.2)	246 (10.4)	2197 (13.7)	
Long COVID at 6 mo				
No. of respondents	10 598	1843	12 441	NA
Present	0	1843 (100)	1843 (14.8)	
Long COVID at 12 mo				
No. of respondents	6327	1135	7462	NA
Present	0	1135 (100)	1135 (15.2)	

Abbreviation: NA, not applicable.

^a^ Other includes Native American, Pacific Islander, or individuals who selected “Other” as race category.

We then reweighted the sample to reflect national sociodemographic distributions, enabling estimates of national point prevalences. Individuals meeting criteria for long COVID represented 13.9% of those who had tested positive for COVID-19 (10.1% of men and 17.9% of women), including 12.6% of Asian adults, 9.7% of Black adults, 15.3% of Hispanic adults, and 15.5% of White adults. In reweighted analysis including all survey participants (eTable 1 in the Supplement), to estimate the proportion of the US adult population who met criteria for current long COVID (ie, point prevalence), these individuals represented 1.7% of US adults; this included 1.3% of men, 2.0% of women, 0.7% of Asian adults, 1.0% of Black adults, 2.0% of Hispanic adults, and 1.8% of White adults.

**Figure 1.**
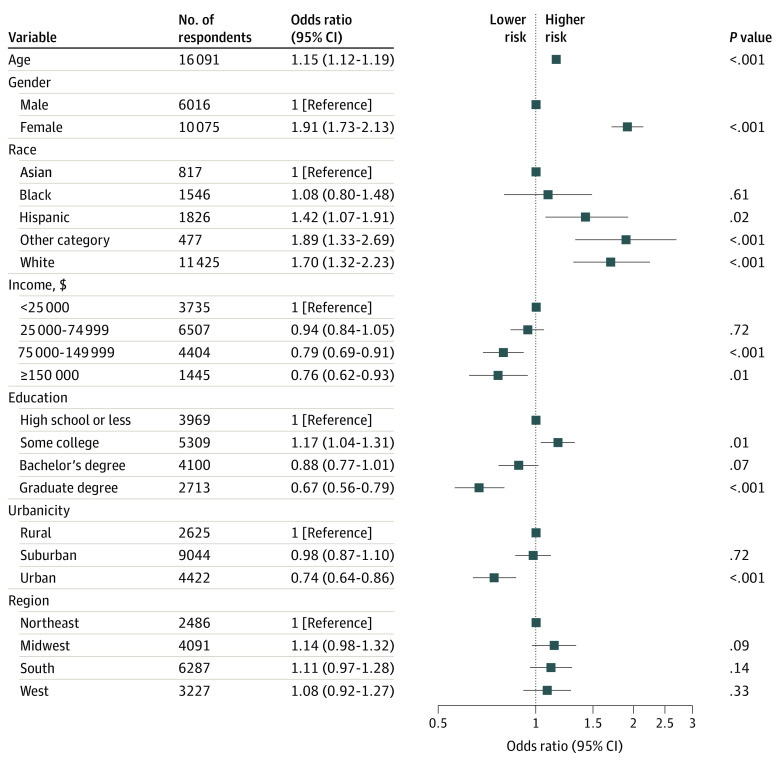
Logistic Regression Model for Development of Long COVID Among Individuals Testing Positive for COVID-19 by Antigen Test or Polymerase Chain Reaction Test

In logistic regression models including sociodemographic features (Figure 1), older age per decade above 40 years (adjusted odds ratio [OR], 1.15; 95% CI, 1.12-1.19) and female gender (adjusted OR, 1.91; 95% CI, 1.73-2.13) were associated with greater risk of persistence; individuals with a graduate education vs high school or less (adjusted OR, 0.67; 95% CI, 0.56-0.79) and urban vs rural residence (adjusted OR, 0.74; 95% CI, 0.64-0.86) were less likely to report persistence. In light of conflicting results from prior investigations, we also examined nonlinear associations of age (eFigure 1 in the Supplement), with maximal liability observed in the age group of 50 to 59 years—compared with the reference group aged 18 to 29 years, the adjusted OR was 2.38 (95% CI, 1.92-2.98).

Table 2 summarizes individual symptoms most commonly reported by survey participants. Fatigue was most common (1232 of 2359 [52.2%]), followed by loss of smell (1031 of 2359 [43.7%]), “brain fog” (952 of 2359 [40.4%]), and shortness of breath (937 of 2359 [39.7%]); 1079 of 2359 participants (45.7%) reported either poor memory or brain fog. Frequencies of individual symptoms differed significantly by gender: women were significantly more likely than men to report loss of smell (832 of 1795 [46.4%] vs 199 of 564 [35.3%]; *P* < .001), cognitive symptoms (874 of 1795 [48.7%] vs 205 of 564 [36.3%]; *P* < .001), anxiety (552 of 1795 [30.8%] vs 126 of 564 [22.3%]; *P* < .001), and sleep disruption (581 of 1795 [32.4%] vs 127 of 564 [22.5%]; *P* < .001). In exploratory analysis, symptom frequencies were generally similar by predominant variant at time of initial illness (eTable 2 in the Supplement), with the exception that anosmia was less frequently reported for infections when the Omicron variant was the predominant variant (Omicron variant, 83 of 246 [33.7%]; Alpha variant, 59 of 147 [40.1%]; Delta variant, 210 of 416 [50.5%]; *P* < .001). In population-weighted estimates, 0.7% (95% CI, 0.7%-0.8%) of sampled US adults reported cognitive symptoms; this sample included 6.1% (95% CI, 5.7%-6.6%) of those with a prior positive COVID-19 test result.

**Table 2.** Frequency of Current Long COVID Symptoms by Gender

Symptom	Individuals, No. (%)			*P* value
	Male (n = 564)	Female (n = 1795)	Total (N = 2359)	
Shortness of breath	230 (40.8)	707 (39.4)	937 (39.7)	.56
Exercise intolerance	161 (28.5)	524 (29.2)	685 (29.0)	.77
Fatigue	267 (47.3)	965 (53.8)	1232 (52.2)	.008
Headache	161 (28.5)	632 (35.2)	793 (33.6)	.003
Loss of smell	199 (35.3)	832 (46.4)	1031 (43.7)	<.001
Brain fog	164 (29.1)	788 (43.9)	952 (40.4)	<.001
Poor memory	120 (21.3)	544 (30.3)	664 (28.1)	<.001
Either brain fog or poor memory	205 (36.3)	874 (48.7)	1079 (45.7)	<.001
Dizziness	92 (16.3)	393 (21.9)	485 (20.6)	.004
Depressed mood	116 (20.6)	434 (24.2)	550 (23.3)	.08
Anxious mood	126 (22.3)	552 (30.8)	678 (28.7)	<.001
Sleep disruption	127 (22.5)	581 (32.4)	708 (30.0)	<.001
Symptom count, mean (SD), No.	3.1 (2.5)	3.9 (2.8)	3.7 (2.7)	<.001

**Figure 2.**
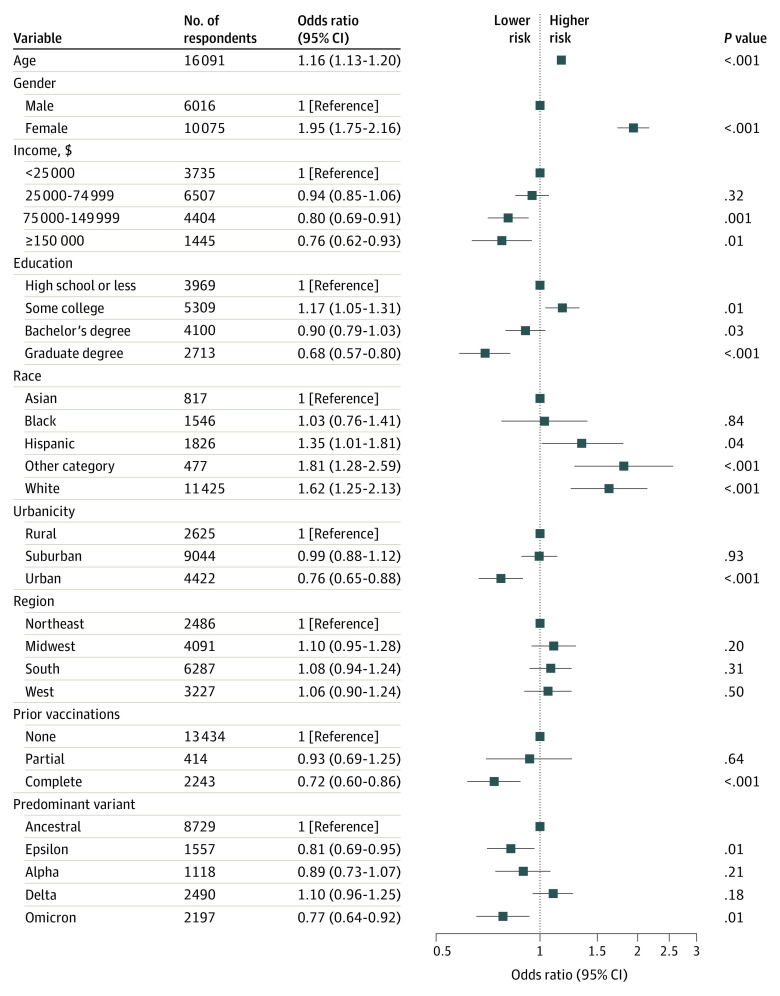
Logistic Regression Model for Development of Long COVID Among Individuals Testing Positive for COVID-19 by Antigen Test or Polymerase Chain Reaction Test, Including Predominant Variant and Vaccination Status at Time of Infection

We next examined the association of predominant variant at time of infection and of vaccination prior to acute illness with risk for long COVID. Compared with ancestral COVID-19, infection during periods when the Epsilon variant (OR, 0.81; 95% CI, 0.69-0.95) or the Omicron variant (OR, 0.77; 95% CI, 0.64-0.92) predominated in the US was associated with diminished likelihood of long COVID (Figure 2). Completion of the primary vaccine series prior to acute illness was associated with diminished risk for long COVID (OR, 0.72; 95% CI, 0.60-0.86). However, partial vaccination (ie, a single vaccination from a 2-vaccine series) was not associated with significant reduction in risk in fully adjusted models (OR, 0.93; 95% CI, 0.69-1.25). A sensitivity analysis excluding infection prior to January 2021, to exclude secular trends or biases arising from inclusion of infection before vaccination was more widely available, yielded similar results (for completion of primary vaccination: OR, 0.73; 95% CI, 0.60-0.88; for partial vaccination: OR, 0.94; 95% CI, 0.69-1.25) (eFigure 2 in the Supplement).

In a sensitivity analysis, when individuals who identified the effect of ongoing symptoms as “not at all” or their only symptom as loss of smell were excluded (n = 354), 2005 of 16 091 participants (12.5%; 12.0% with US population weighting) met diagnostic criteria (eTable 3 in the Supplement). Regression models yielded similar results for sociodemographic features (eFigure 3 in the Supplement) and greater numeric magnitude of benefit associated with prior vaccination (eFigure 4 in the Supplement) (for complete vaccination: OR, 0.69; 95% CI, 0.57-0.84).

## Discussion

In this cross-sectional study of a cohort of 16 091 adults surveyed between February 2021 and July 2022 in all 50 states in the US and the District of Columbia, we estimated that 14.7% of those who reported a positive COVID-19 test result more than 2 months previously continued to describe symptoms that they associated with acute infection, or 13.9% after reweighting to reflect the US adult population. These point prevalence estimates were similar when the cohort was restricted to those whose acute illness was 6 and 12 months in the past.

Our results are broadly similar to those previously reported among nonhospitalized cohorts. In a study using app-based symptom recording for 4182 patients with COVID-19, only 4.5% reported symptoms for more than 8 weeks, broadly similar to our results—as in the inpatient cohorts, fatigue was among the most common symptoms, along with dyspnea and headache.^13^ Our results are consistent with that app-based study in identifying age and female gender as factors associated with risk, even though our sampling frame is markedly different. Our design more closely resembles that of a very large-scale UK survey, which found the greatest risk for persistence among female respondents and younger respondents.^6^ More broadly, the differences among these 3 studies, which all used self-report, may reflect differences in ascertainment and question design and may indicate the importance of multiple convergent methods to characterize long COVID. The associations with income, educational level, and race and ethnicity that we identified in our sample highlight the importance of considering these features in understanding the differential longer-term, as well as shorter-term, outcomes of infection.

Two recent studies directly examined the question of protection afforded by prior vaccination, using different designs. One study examined more than 33 000 previously vaccinated individuals with breakthrough COVID-19 infection from Veterans Affairs electronic health records.^14^ The Veterans Affairs population may not fully reflect the general adult population in the US, and coded clinical data may be less sensitive to symptoms than narrative notes or patient-reported symptoms.^9^ Still, despite these differences, the approximately 24% reduction in odds of long COVID that we observed after a single vaccination (approximately 33% when applying a stricter definition of long COVID) does approximate the 15% reduction in hazard of long COVID in that study.^14^

A complementary study drew on self-report from more than 1 million users of a UK COVID-19 symptom app, including approximately 8400 users who experienced COVID-19 infection after at least 1 vaccine dose.^15^ In that study, no apparent protective association of an initial vaccine dose with symptoms beyond 28 days was detected (OR, 1.03; 95% CI, 0.85-1.24), consistent with our findings after a single dose, although marked protection was observed after a second dose (OR, 0.51; 95% CI, 0.32-0.82).

### Limitations and Strengths

This study has some limitations. First, because this study used preempaneled respondents in a nonprobability design, we cannot reliably calculate the response rate; as such, nonresponse bias cannot be estimated. However, in other domains, these nonprobability surveys have closely mirrored results from more traditional designs,^24^ and prior work with this survey found results that closely approximate estimates obtained using other methods, including probability polls and administrative data.^25,26^ Furthermore, our cross-sectional design does not allow for a more precise estimate of symptom persistence and relies on participant recall in some cases nearly 1 year after initial illness. In particular, we cannot exclude the possibility that some individuals who previously experienced long COVID symptoms had recovered by the time of the survey, although the stability of our estimates when samples were restricted to greater follow-up periods since acute infection suggests that this is less likely. Misclassification of individuals who previously had long COVID but recovered at the time of the survey should bias our results toward smaller estimates of effect, such that any associations we identify may actually represent conservative estimates. Conversely, absent measures of symptom frequency among individuals without prior COVID-19, we cannot estimate the extent to which apparent long COVID symptoms would be identified as a consequence of other illnesses. Lacking detailed assessment of respondents’ medical history, we also cannot examine the associations of comorbid medical illness or acuity of acute illness with risk for long COVID, which could explain some of the observed associations. Finally, we relied on self-report of symptoms rather than objective physiological or cognitive measures. As such, our results must be seen as complementing, rather than replacing, analyses using administrative claims^14^ or electronic health records. Prospective studies will be necessary to confirm our results; the National Institutes of Health RECOVER (Researching COVID to Enhance Recovery) study, for example, will be valuable in providing systematic and objective measurement of sequelae.^27^

Despite these limitations, we also emphasize the strengths of this systematic assessment, namely, that by design it should be more representative than other single-cohort studies because it captures individuals drawn from every state. Moreover, because the survey is not specifically aimed at individuals with COVID-19 or symptom persistence, it may be less biased toward those with a greater interest in long-term symptoms than (for example) symptom tracking applications.^13^ That is, because recruitment materials did not specify COVID-19 or persistence, our results are less likely to reflect individuals with greater interest in COVID-19 persistence. At the other extreme, our approach is less likely to overestimate prevalence than investigations based solely on artifacts of clinical care.

A key question for further investigation will be the differences by race and ethnicity in the prevalence of long COVID that we observed, even after accounting for a range of sociodemographic correlates. These differences cannot be explained by a lack of access to COVID-19 testing because our outcome definition was contigent on obtaining such a test. The finding that greater educational levels, greater income, and urban vs rural setting are associated with diminished long COVID risk highlights the importance of accounting for nonbiological associations in understanding this phenomenon, a limitation of prior investigations. Finally, the suggestion that rates of long COVID may vary by predominant variant at time of infection also merits further investigation because it may help to inform efforts to understand the mechanisms underlying the development of this syndrome.

## Conclusions

In aggregate, the results of this cross-sectional study provide an estimate of the mean point prevalence of long COVID in a large, representative population sample of individuals in the United States, complementing studies using administrative claims, electronic health records, or COVID-19–focused self-report apps and surveys. They support the potential protective association of vaccination in reducing but not eliminating long COVID risk. If confirmed in prospective studies, these results may facilitate risk stratification, with a goal of early intervention to minimize the effect of long COVID, and could contribute to efforts to prevent this syndrome altogether.

## Notes

Supplement. **eTable 1.** Point Prevalences of Long COVID-19 Among Individuals Testing Positive for COVID-19 by Antigen Test or PCR, and Among All Surveyed US Adults, Weighted to Reflect US Adult Population **eFigure 1.** Logistic Regression Model for Development of Long COVID-19 Among Individuals Testing Positive for COVID-19 by Antigen Test or PCR, With Age by Decade **eTable 2.** Long COVID-19 Symptom Frequencies by Predominant Variant at Time of Initial Illness **eTable 3.** Characteristics of Individuals Who Tested Positive for COVID-19 by Antigen Test or PCR at Least Two Months Prior to Survey Date, Who Did or Did Not Experience Long COVID-19 Symptoms by More Stringent Definition **eFigure 2.** Logistic Regression Model for Development of Long COVID-19 Among Individuals Testing Positive for COVID-19 by Antigen Test or PCR, With Age by Decade, Limited to Individuals With Index Infection January 2021 or Later **eFigure 3.** Logistic Regression Model for Development of More Stringently Defined Long COVID-19 Among Individuals Testing Positive for COVID-19 by Antigen Test or PCR **eFigure 4.** Logistic Regression Model for Development of Long COVID-19 by More Stringent Definition Among Individuals Testing Positive for COVID-19 by Antigen Test or PCR, Including Predominant Variant and Vaccination Status
