# Supplementary material for: Prevalence and Correlates of Long COVID Symptoms Among US Adults
Source: JAMA Netw Open. 2022 Oct 27;5(10):e2238804. doi: 10.1001/jamanetworkopen.2022.38804 (PMC9614581; doi:10.1001/jamanetworkopen.2022.38804)
Supplement: Supplement. — eTable 1. Point Prevalences of Long COVID-19 Among Individuals Testing Positive for COVID-19 by Antigen Test or PCR, and Among All Surveyed US Adults, Weighted to Reflect US Adult Population eFigure 1. Logistic Regression Model for Development of Long COVID-19 Among Individuals Testing Positive for COVID-19 by Antigen Test or PCR, With Age by Decade eTable 2. Long COVID-19 Symptom Frequencies by Predominant Variant at Time of Initial Illness eTable 3. Characteristics of Individuals Who Tested Positive for COVID-19 by Antigen Test or PCR at Least Two Months Prior to Survey Date, Who Did or Did Not Experience Long COVID-19 Symptoms by More Stringent Definition eFigure 2. Logistic Regression Model for Development of Long COVID-19 Among Individuals Testing Positive for COVID-19 by Antigen Test or PCR, With Age by Decade, Limited to Individuals With Index Infection January 2021 or Later eFigure 3. Logistic Regression Model for Development of More Stringently Defined Long COVID-19 Among Individuals Testing Positive for COVID-19 by Antigen Test or PCR eFigure 4. Logistic Regression Model for Development of Long COVID-19 by More Stringent Definition Among Individuals Testing Positive for COVID-19 by Antigen Test or PCR, Including Predominant Variant and Vaccination Status [file jamanetwopen-e2238804-s001.pdf]

Supplementary Online Content

Perlis RH, Santillana M, Ognyanova K, et al. Prevalence and correlates of long COVID symptoms among US adults. *JAMA Netw Open.* 2022;5(10):e2238804. doi:10.1001/jamanetworkopen.2022.38804

eTable 1. Point Prevalences of Long COVID-19 Among Individuals Testing Positive for COVID-19 by Antigen Test or PCR, and Among All Surveyed US Adults, Weighted to Reflect US Adult Population
eFigure 1. Logistic Regression Model for Development of Long COVID-19 Among Individuals Testing Positive for COVID-19 by Antigen Test or PCR, With Age by Decade
eTable 2. Long COVID-19 Symptom Frequencies by Predominant Variant at Time of Initial Illness
eTable 3. Characteristics of Individuals Who Tested Positive for COVID-19 by Antigen Test or PCR at Least Two Months Prior to Survey Date, Who Did or Did Not Experience Long COVID-19 Symptoms by More Stringent Definition
eFigure 2. Logistic Regression Model for Development of Long COVID-19 Among Individuals Testing Positive for COVID-19 by Antigen Test or PCR, With Age by Decade, Limited to Individuals With Index Infection January 2021 or Later
eFigure 3. Logistic Regression Model for Development of More Stringently Defined Long COVID-19 Among Individuals Testing Positive for COVID-19 by Antigen Test or PCR
eFigure 4. Logistic Regression Model for Development of Long COVID-19 by More Stringent Definition Among Individuals Testing Positive for COVID-19 by Antigen Test or PCR, Including Predominant Variant and Vaccination Status

This supplementary material has been provided by the authors to give readers additional information about their work.

© 2022 Perlis RH et al. *JAMA Network Open.*

eTable 1. Point Prevalences of Long COVID-19 Among Individuals Testing Positive for COVID-19 by Antigen Test or PCR, and Among All Surveyed US Adults, Weighted to Reflect US Adult Population

	**Proportion of *COVID positive* US adults**				**Proportion of all US adults**		
	**estimate**	**[95% CI**	**]**		**estimate**	**[95% CI**	**]**
**Overall**	13.9%	13.3%	14.6%		1.7%	1.6%	1.7%
**Gender**							
Male	10.2%	9.3%	11.1%		1.3%	1.2%	1.4%
Female	17.9%	17.0%	18.8%		2.0%	1.9%	2.1%
**Age**							
18-29	10.2%	8.3%	12.4%		1.4%	1.1%	1.7%
30-39	10.8%	9.8%	12.1%		1.6%	1.4%	1.8%
40-49	12.6%	11.6%	13.6%		1.8%	1.7%	1.9%
50-59	20.2%	18.2%	22.4%		2.2%	2.0%	2.5%
60-69	18.3%	16.1%	20.7%		1.5%	1.3%	1.7%
70+	14.3%	11.6%	17.4%		1.0%	0.8%	1.2%
**Race and Ethnicity**							
Asian	7.5%	5.7%	10.0%		0.7%	0.5%	0.9%
Black	9.7%	8.2%	11.5%		1.0%	0.9%	1.2%
Hispanic	12.6%	10.8%	14.5%		2.0%	1.7%	2.4%
White	15.5%	14.7%	16.3%		1.8%	1.7%	1.9%
Other	15.3%	12.0%	19.2%		1.7%	1.3%	2.1%

© 2022 Perlis RH et al. *JAMA Network Open.*

eFigure 1. Logistic Regression Model for Development of Long COVID-19 Among Individuals Testing Positive for COVID-19 by Antigen Test or PCR, With Age by Decade

Variable		N	Odds ratio	p
**Age**	18−29	1076	Reference	
	30−39	3629	1.12 (0.91, 1.40)	0.295
	40−49	7049	1.49 (1.22, 1.84)	<0.001
	50−59	2056	2.38 (1.92, 2.98)	<0.001
	60−69	1512	1.96 (1.55, 2.48)	<0.001
	70+	769	1.64 (1.24, 2.17)	<0.001
**Gender**	Male	6016	Reference	
	Female	10075	1.88 (1.70, 2.09)	<0.001
**Race**	Asian	817	Reference	
	Black	1546	1.06 (0.78, 1.44)	0.717
	Hispanic	1826	1.40 (1.06, 1.89)	0.022
	Other category	477	1.86 (1.31, 2.65)	<0.001
	White	11425	1.69 (1.31, 2.22)	<0.001
**Income**	Less than $25,000	3735	Reference	
	$25,000 to $74,999	6507	0.94 (0.84, 1.05)	0.270
	$75,000 to $149,999	4404	0.78 (0.68, 0.89)	<0.001
	$150,000 or more	1445	0.73 (0.59, 0.90)	0.003
**Education**	High School or Less	3969	Reference	
	Some College	5309	1.16 (1.04, 1.30)	0.009
	Bachelor's Degree	4100	0.87 (0.76, 1.00)	0.048
	Graduate Degree	2713	0.66 (0.56, 0.79)	<0.001
**Urbanicity**	Rural	2625	Reference	
	Suburban	9044	0.99 (0.88, 1.11)	0.834
	Urban	4422	0.75 (0.65, 0.87)	<0.001
**Region**	Northeast	2486	Reference	
	Midwest	4091	1.13 (0.98, 1.32)	0.100
	South	6287	1.11 (0.97, 1.28)	0.146
	West	3227	1.08 (0.92, 1.27)	0.321

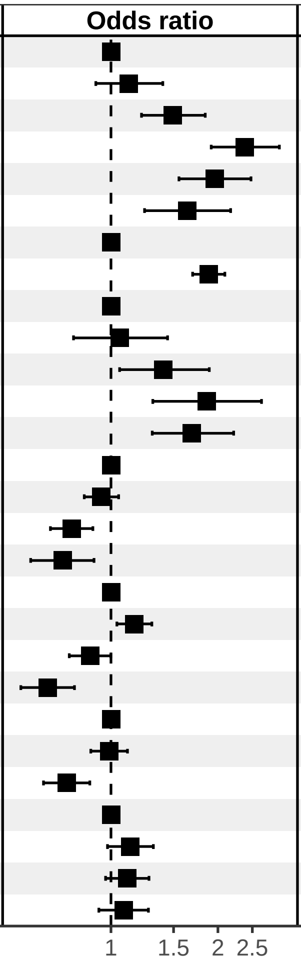

© 2022 Perlis RH et al. *JAMA Network Open.*

eTable 2. Long COVID-19 Symptom Frequencies by Predominant Variant at Time of Initial Illness

Supplemental Table. Long COVID symptoms, by predominant variant

	alpha (N=147)	ancestral (N=1344)	delta (N=416)	epsilon (N=206)	omicron (N=246)	Total (N=2359)	p value
**Shortness of breath**	58 (39.5%)	554 (41.2%)	149 (35.8%)	74 (35.9%)	102 (41.5%)	937 (39.7%)	0.243
**Exercise intolerance**	37 (25.2%)	387 (28.8%)	129 (31.0%)	60 (29.1%)	72 (29.3%)	685 (29.0%)	0.755
**Fatigue**	78 (53.1%)	708 (52.7%)	214 (51.4%)	92 (44.7%)	140 (56.9%)	1232 (52.2%)	0.128
**Headache**	49 (33.3%)	457 (34.0%)	131 (31.5%)	68 (33.0%)	88 (35.8%)	793 (33.6%)	0.829
**Loss of smell**	59 (40.1%)	590 (43.9%)	210 (50.5%)	89 (43.2%)	83 (33.7%)	1031 (43.7%)	< 0.001
**Brain fog**	51 (34.7%)	548 (40.8%)	165 (39.7%)	85 (41.3%)	103 (41.9%)	952 (40.4%)	0.655
**Poor memory**	33 (22.4%)	381 (28.3%)	114 (27.4%)	66 (32.0%)	70 (28.5%)	664 (28.1%)	0.399
**(Either cognitive symptom)**	58 (39.5%)	623 (46.4%)	184 (44.2%)	97 (47.1%)	117 (47.6%)	1079 (45.7%)	0.493
**Dizziness**	33 (22.4%)	269 (20.0%)	79 (19.0%)	44 (21.4%)	60 (24.4%)	485 (20.6%)	0.480
**Depressed mood**	34 (23.1%)	319 (23.7%)	94 (22.6%)	49 (23.8%)	54 (22.0%)	550 (23.3%)	0.970
**Anxious mood**	35 (23.8%)	400 (29.8%)	120 (28.8%)	62 (30.1%)	61 (24.8%)	678 (28.7%)	0.344
**Sleep disruption**	40 (27.2%)	409 (30.4%)	125 (30.0%)	59 (28.6%)	75 (30.5%)	708 (30.0%)	0.928
							0.805
**Mean (SD) symptom count**	3.449 (2.758)	3.737 (2.755)	3.678 (2.708)	3.631 (2.859)	3.691 (2.627)	3.694 (2.742)	

© 2022 Perlis RH et al. *JAMA Network Open.*

eTable 3. Characteristics of Individuals Who Tested Positive for COVID-19 by Antigen Test or PCR at Least Two Months Prior to Survey Date, Who Did or Did Not Experience Long COVID-19 Symptoms by More Stringent Definition

	No Long COVID (N=14086)	Long COVID (N=2005)	Total (N=16091)	p value
Gender				< 0.001
Male	5528 (39.2%)	488 (24.3%)	6016 (37.4%)	
Female	8558 (60.8%)	1517 (75.7%)	10075 (62.6%)	
Age (years)				< 0.001
Mean (SD)	39.99 (15.19)	44.17 (15.07)	40.51 (15.23)	
Education				< 0.001
High School or Less	3440 (24.4%)	529 (26.4%)	3969 (24.7%)	
Some College	4480 (31.8%)	829 (41.3%)	5309 (33.0%)	
Bachelor’s Degree	3656 (26.0%)	444 (22.1%)	4100 (25.5%)	
Graduate Degree	2510 (17.8%)	203 (10.1%)	2713 (16.9%)	
Income				< 0.001
Less than $25,000	3174 (22.5%)	561 (28.0%)	3735 (23.2%)	
$25,000 to $74,999	5602 (39.8%)	905 (45.1%)	6507 (40.4%)	
$75,000 to $149,999	3995 (28.4%)	409 (20.4%)	4404 (27.4%)	
$150,000 or more	1315 (9.3%)	130 (6.5%)	1445 (9.0%)	
Race				< 0.001
White	9879 (70.1%)	1546 (77.1%)	11425 (71.0%)	
Black	1631 (11.6%)	195 (9.7%)	1826 (11.3%)	
Hispanic	1408 (10.0%)	138 (6.9%)	1546 (9.6%)	
Asian	764 (5.4%)	53 (2.6%)	817 (5.1%)	
Other	404 (2.9%)	73 (3.6%)	477 (3.0%)	
Urbanicity				< 0.001
rural	2219 (15.8%)	406 (20.2%)	2625 (16.3%)	
suburban	7833 (55.6%)	1211 (60.4%)	9044 (56.2%)	
urban	4034 (28.6%)	388 (19.4%)	4422 (27.5%)	
Region				< 0.001
Northeast	2224 (15.8%)	262 (13.1%)	2486 (15.4%)	
Midwest	3533 (25.1%)	558 (27.8%)	4091 (25.4%)	
South	5468 (38.8%)	819 (40.8%)	6287 (39.1%)	
West	2861 (20.3%)	366 (18.3%)	3227 (20.1%)	
Vaccination status				< 0.001
Unvaccinated	11692 (83.0%)	1742 (86.9%)	13434 (83.5%)	
Partial	362 (2.6%)	52 (2.6%)	414 (2.6%)	
Complete	2032 (14.4%)	211 (10.5%)	2243 (13.9%)	
Predominant variant				< 0.001

© 2022 Perlis RH et al. *JAMA Network Open.*

	No Long COVID (N=14086)	Long COVID (N=2005)	Total (N=16091)	p value
alpha	992 (7.0%)	126 (6.3%)	1118 (6.9%)	
ancestral	7590 (53.9%)	1139 (56.8%)	8729 (54.2%)	
delta	2140 (15.2%)	350 (17.5%)	2490 (15.5%)	
epsilon	1386 (9.8%)	171 (8.5%)	1557 (9.7%)	
omicron	1978 (14.0%)	219 (10.9%)	2197 (13.7%)	

© 2022 Perlis RH et al. *JAMA Network Open.*

eFigure 2. Logistic Regression Model for Development of Long COVID-19 Among Individuals Testing Positive for COVID-19 by Antigen Test or PCR, With Age by Decade, Limited to Individuals With Index Infection January 2021 or Later

Variable		N	Odds ratio	p
**Age**		7362	1.16 (1.11, 1.21)	<0.001
**Gender**	Male	2575	Reference	
	Female	4787	1.89 (1.62, 2.22)	<0.001
**Income**	Less than $25,000	1832	Reference	
	$25,000 to $74,999	3109	0.91 (0.77, 1.07)	0.258
	$75,000 to $149,999	1855	0.78 (0.63, 0.96)	0.018
	$150,000 or more	566	0.68 (0.48, 0.94)	0.022
**Education**	High School or Less	1962	Reference	
	Some College	2492	1.24 (1.05, 1.47)	0.011
	Bachelor's Degree	1880	0.88 (0.71, 1.07)	0.206
	Graduate Degree	1028	0.77 (0.59, 1.01)	0.059
**Race**	Asian	431	Reference	
	Black	734	1.26 (0.79, 2.05)	0.341
	Hispanic	790	1.47 (0.94, 2.37)	0.105
	Other category	218	2.27 (1.32, 3.94)	0.003
	White	5189	2.01 (1.35, 3.12)	<0.001
**Urbanicity**	Rural	1240	Reference	
	Suburban	4241	0.99 (0.83, 1.18)	0.871
	Urban	1881	0.70 (0.55, 0.88)	0.003
**Region**	Northeast	1198	Reference	
	Midwest	1744	1.14 (0.91, 1.43)	0.275
	South	2967	1.12 (0.91, 1.39)	0.285
	West	1453	1.11 (0.87, 1.41)	0.423
**Prior vaccinations**	None	4705	Reference	
	Partial	414	0.94 (0.69, 1.25)	0.659
	Complete	2243	0.73 (0.60, 0.88)	<0.001
**Predominant Variant**	epsilon	1557	Reference	
	alpha	1118	1.09 (0.86, 1.38)	0.461
	delta	2490	1.33 (1.09, 1.61)	0.004
	omicron	2197	0.94 (0.75, 1.18)	0.595

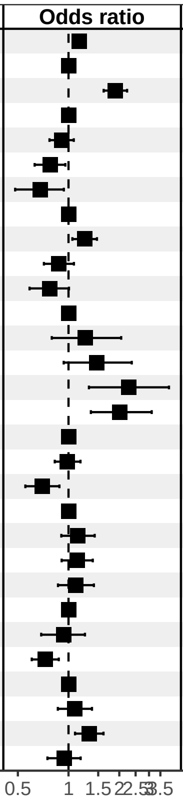

© 2022 Perlis RH et al. *JAMA Network Open.*

eFigure 3. Logistic Regression Model for Development of More Stringently Defined Long COVID-19 Among Individuals Testing Positive for COVID-19 by Antigen Test or PCR

Variable		N	Odds ratio	p
**Age**		16091	1.18 (1.14, 1.21)	<0.001
**Gender**	Male	6016	Reference	
	Female	10075	1.83 (1.64, 2.05)	<0.001
**Race**	Asian	817	Reference	
	Black	1546	1.07 (0.77, 1.51)	0.688
	Hispanic	1826	1.48 (1.08, 2.05)	0.017
	Other category	477	2.01 (1.38, 2.95)	<0.001
	White	11425	1.74 (1.31, 2.35)	<0.001
**Income**	Less than $25,000	3735	Reference	
	$25,000 to $74,999	6507	0.91 (0.81, 1.02)	0.106
	$75,000 to $149,999	4404	0.70 (0.61, 0.81)	<0.001
	$150,000 or more	1445	0.77 (0.62, 0.95)	0.016
**Education**	High School or Less	3969	Reference	
	Some College	5309	1.19 (1.05, 1.34)	0.005
	Bachelor's Degree	4100	0.92 (0.80, 1.06)	0.274
	Graduate Degree	2713	0.70 (0.58, 0.84)	<0.001
**Urbanicity**	Rural	2625	Reference	
	Suburban	9044	0.96 (0.85, 1.09)	0.536
	Urban	4422	0.73 (0.62, 0.86)	<0.001
**Region**	Northeast	2486	Reference	
	Midwest	4091	1.16 (0.99, 1.36)	0.074
	South	6287	1.14 (0.98, 1.33)	0.087
	West	3227	1.11 (0.94, 1.32)	0.226

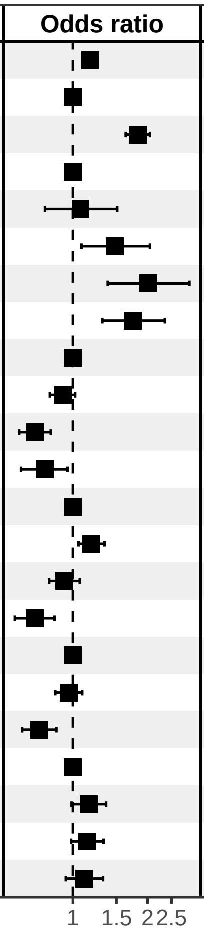

© 2022 Perlis RH et al. *JAMA Network Open.*

eFigure 4. Logistic Regression Model for Development of Long COVID-19 by More Stringent Definition Among Individuals Testing Positive for COVID-19 by Antigen Test or PCR, Including Predominant Variant and Vaccination Status

Variable		N	Odds ratio		p
**Age**		16091		1.19 (1.15, 1.22)	<0.001
**Gender**	Male	6016		Reference	
	Female	10075		1.85 (1.66, 2.08)	<0.001
**Income**	Less than $25,000	3735		Reference	
	$25,000 to $74,999	6507		0.91 (0.81, 1.03)	0.129
	$75,000 to $149,999	4404		0.71 (0.61, 0.82)	<0.001
	$150,000 or more	1445		0.77 (0.62, 0.95)	0.018
**Education**	High School or Less	3969		Reference	
	Some College	5309		1.19 (1.06, 1.35)	0.004
	Bachelor's Degree	4100		0.95 (0.82, 1.09)	0.441
	Graduate Degree	2713		0.71 (0.59, 0.86)	<0.001
**Race**	Asian	817		Reference	
	Black	1546		1.02 (0.74, 1.44)	0.887
	Hispanic	1826		1.41 (1.03, 1.95)	0.036
	Other category	477		1.94 (1.33, 2.84)	<0.001
	White	11425		1.67 (1.26, 2.25)	<0.001
**Urbanicity**	Rural	2625		Reference	
	Suburban	9044		0.98 (0.86, 1.11)	0.699
	Urban	4422		0.74 (0.63, 0.87)	<0.001
**Region**	Northeast	2486		Reference	
	Midwest	4091		1.12 (0.96, 1.32)	0.155
	South	6287		1.11 (0.95, 1.29)	0.189
	West	3227		1.09 (0.92, 1.30)	0.337
**Prior vaccinations**	None	13434		Reference	
	Partial	414		0.97 (0.70, 1.31)	0.823
	Complete	2243		0.69 (0.57, 0.84)	<0.001
**Predominant Variant**	ancestral	8729		Reference	
	epsilon	1557		0.80 (0.67, 0.95)	0.011
	alpha	1118		0.91 (0.74, 1.10)	0.337
	delta	2490		1.09 (0.94, 1.26)	0.234
	omicron	2197		0.84 (0.69, 1.02)	0.079

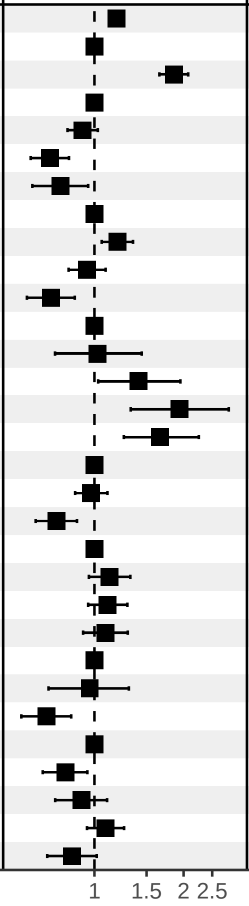

© 2022 Perlis RH et al. *JAMA Network Open.*
